# Associations between prediagnostic blood glucose levels, diabetes, and glioma

**DOI:** 10.1038/s41598-017-01553-2

**Published:** 2017-05-03

**Authors:** Judith Schwartzbaum, Michael Edlinger, Victoria Zigmont, Pär Stattin, Grzegorz A. Rempala, Gabriele Nagel, Niklas Hammar, Hanno Ulmer, Bernhard Föger, Göran Walldius, Jonas Manjer, Håkan Malmström, Maria Feychting

**Affiliations:** 1 Division of Epidemiology, College of Public Health, Ohio State University, Columbus, Ohio 43210 United States of America; 2 Comprehensive Cancer Center, Ohio State University, Columbus, Ohio 43210 United States of America; 3 Department of Medical Statistics, Informatics, and Health Economics, Medical University, Innsbruck, Austria; 4 Department of Public Health, Southern Connecticut State University, New Haven, CT 06515 United States of America; 5 Department of Surgical and Perioperative Sciences, Urology and Andrology, Umeå University, Umeå, Sweden; 6 Division of Biostatistics, College of Public Health, Ohio State University, Columbus, Ohio 43210 United States of America; 7 Mathematical Biosciences Institute, Columbus, Ohio 43210 United States of America; 8 Institute of Epidemiology and Medical Biometry, Ulm University, Ulm, Germany; 9 Agency for Preventive and Social Medicine, Bregenz, Austria; 10 Medical Evidence & Observational Research, Global Medical Affairs, Astra Zeneca R&D, Mölndal, 43150 Sweden; 11 Unit of Epidemiology, Institute of Environmental Medicine, Karolinska Institutet, SE-17177 Stockholm, Sweden; 12 Unit of Cardiovascular Epidemiology, Institute of Environmental Medicine, Karolinska Institutet, SE-17177 Stockholm, Sweden; 13 Department of Surgery, Skåne University Hospital, Malmö, Sweden

## Abstract

Previous literature indicates that pre-diagnostic diabetes and blood glucose levels are inversely related to glioma risk. To replicate these findings and determine whether they could be attributed to excess glucose consumption by the preclinical tumour, we used data from the Apolipoprotein MOrtality RISk (AMORIS) (n = 528,580) and the Metabolic syndrome and Cancer project (Me-Can) cohorts (n = 269,365). We identified individuals who were followed for a maximum of 15 years after their first blood glucose test until glioma diagnosis, death, emigration or the end of follow-up. Hazard ratios (HRs), 95% confidence intervals (CIs) and their interactions with time were estimated using Cox time-dependent regression. As expected, pre-diagnostic blood glucose levels were inversely related to glioma risk (AMORIS, *P*_trend_ = 0.002; Me-Can, *P*_trend_ = 0.04) and pre-diagnostic diabetes (AMORIS, HR = 0.30, 95% CI 0.17 to 0.53). During the year before diagnosis, blood glucose was inversely associated with glioma in the AMORIS (HR = 0.78, 95% CI 0.66 to 0.93) but not the Me-Can cohort (HR = 0.99, 95% CI 0.63 to 1.56). This AMORIS result is consistent with our hypothesis that excess glucose consumption by the preclinical tumour accounts for the inverse association between blood glucose and glioma. We discuss additional hypothetical mechanisms that may explain our paradoxical findings.

## Introduction

Glioma is a heterogeneous primary brain tumour for which there is no treatment that ensures long-term survival. Patients diagnosed with the most common adult form of this tumour, glioblastoma, survive on average only 14 months^1^. Symptoms usually occur only three months before diagnosis^2^; it is therefore important that indicators of the preclinical tumour be identified before the onset of symptoms.

Diabetes is a metabolic disease resulting from defects in insulin production leading to the inability of cells to use glucose and thus to elevated blood glucose levels. Although diabetes appears to increase the risk of most malignant tumours^3^, 11 out of 16 previous studies (*e.g*. refs 4–7) found an inverse association between diabetes and glioma or all types of brain tumours. A recent meta-analysis^8^ confirmed this inverse relationship. However, a recent cohort study of pre-diagnostic diabetes and cancer at all sites, in 2.3 million Israeli adults^9^, found an increased risk of malignant brain tumours among people diagnosed with diabetes.

People with diabetes may be diagnosed at different times during the course of the disease and this diagnosis is usually followed by treatments that may affect associations with subsequent glioma risk. We therefore focus the present study on associations between pre-diagnostic blood glucose levels and glioma risk as blood glucose levels may be measured routinely and are not necessarily followed by treatment. There are two previous studies reporting inverse associations between pre-diagnostic circulating glucose levels and glioma risk^7, 10^. Our study differs from this previous work because we concentrate on pre-diagnostic blood glucose and evaluate interactions between its levels and time from blood collection to brain tumour diagnosis and age at brain tumour diagnosis. In addition, we examined our data for the presence of competing risks.

Our reason for estimating the modifying effects of time before tumour diagnosis is as follows. If the nascent glioma is responsible for the association between blood glucose level and glioma, we would expect this association to vary with the proximity of blood collection to time of glioma diagnosis. Specifically, we propose that elevated consumption of blood glucose by the preclinical tumour may account for the apparent reduction of glioma risk among people with diabetes or elevated blood glucose levels. Cancer cells generate energy using aerobic glycolysis rather than oxidative phosphorylation used by non-proliferating cells. Aerobic glycolysis is less efficient than oxidative phosphorylation generating only two ATPs per molecule of glucose while oxidative phosphorylation generates 36 ATPs. Therefore, in general, cancer cells require more glucose than do non-proliferating cells. This phenomenon was first reported by Otto Warburg and is therefore referred to as the Warburg effect^11^. There is evidence of aerobic glycolysis in glioma^12–15^. Possibly consistent with this increased need for glucose, Seyfried *et al*.^16^ show that growth of astrocytoma in a mouse model is a direct function of blood glucose levels. Sturrock *et al*.^17^ developed a mathematical model describing this process. If the preclinical tumour reduces circulating blood glucose levels then one would expect an inverse association to be strongest near the time of glioma diagnosis^17^.

Our rationale for evaluating age at diagnosis as a modifying factor is based on results of a previous AMORIS study of pre-diagnostic serum glucose levels and prostate cancer risk^18^. In this study, Van Hemelrijck *et al*. found that the inverse association between blood glucose levels and prostate cancer diagnosis may be attributable, in part, to the phenomenon of competing risks^19^. That is, because elevated blood glucose levels increase the risk of death from both cardiovascular disease^20^ and cancer^21^, people with hyperglycaemia (≥5.6 mmol/L) would be expected to have higher all-cause mortality rates than would people with normal blood glucose levels. Thus people with hyperglycaemia are selected out of the population at risk of glioma making etiological inferences based on comparisons of the prevalence of hyperglycaemia between people with and without glioma invalid. To determine whether this potential source of selection bias is present, we estimated the modifying effects of age at diagnosis on the association between blood glucose levels and glioma. Competing risk bias would be expected to increase with age as do all-cause mortality rates. To further evaluate our data for the presence of competing risks, we used the sub-distribution hazards model^22, 23^ which is a more formal method of identifying competing risks.

## Results

In Table 1 the relative proportions of male and female total glioma cases differ between the cohorts (in AMORIS 65% are men; in Me-Can 52%) as does fasting status (in AMORIS 61% of blood was drawn when total glioma cases were fasting; in Me-Can all blood samples were collected from fasting participants). The remaining descriptive characteristics of both high grade glioma (anaplastic astrocytoma and glioblastoma) and total glioma in the two cohorts are similar. For example, ages at the time of the initial blood test and the time of total glioma diagnosis are almost the same among total glioma patients in the two cohorts (AMORIS: ages 50 and 57 years respectively; Me-Can: ages 50 and 58 years, respectively) resulting in the same median periods of observation among cases (seven years) in both cohorts. Although non-cases have a longer median observation time in the AMORIS (15 years) than in the Me-Can cohort (12 years), total glioma incidence rates are similar. The incidence rate in the AMORIS cohort is 7.6/100,000 person years, while that in the Me-Can cohort is 6.4/100,000 person years.

**Table 1:** **C**ohort characteristics by glioma grade.

Variable	AMORIS^1^ cohort			Me-Can^2^ cohort		
	High Grade Glioma^3^ (n = 476)	Total Glioma (n = 604)	No Glioma (n = 527,976)	High Grade Glioma^3^ (n = 164)	Total Glioma (n = 208)	No Glioma (n = 269,157)
**Gender (n, %)**						
Men	301 (63%)	390 (65%)	283,150 (54%)	90 (55%)	108 (52%)	132,767 (49%)
Women	175 (37%)	214 (35%)	244,826 (46%)	74 (45%)	100 (48%)	136,390 (51%)
**Fasting Status (n, %)**						
Fasting	296 (62%)	366 (61%)	296,850 (56%)	164 (100%)	208 (100%)	269,157 (100%)
Non-Fasting	180 (38%)	238 (39%)	231,126 (44%)	—	—	—
**Demographic (years)**						
Median age at lab test (IQR^4^)	51 (43; 58)	50 (40; 57)	44 (33; 54)	50 (46; 59)	50 (45; 59)	44 (34; 53)
Median age at event or censoring^5^ (IQR^4^)	59 (50; 65)	57 (48; 64)	58 (48; 68)	58 (53; 65)	58 (51; 65)	55 (44; 64)
Median time in study (IQR^4^)	7 (4; 11)	7 (3; 11)	15 (15; 15)	7 (4; 10)	7 (4; 10)	12 (7; 15)
Median year lab test (IQR^4^)	1988 (1986; 1992)	1988 (1986; 1992)	1989 (1986; 1992)	1990 (1987; 1993)	1990 (1988; 1993)	1992 (1989; 1997)
**Serum Glucose (mmol/L)**						
Median blood glucose (IQR^4^)	5.0 (4.9; 5.1)	5.0 (4.9; 5.1)	5.0 (5.0; 5.0)	4.9 (4.4; 5.4)	4.9 (4.4; 5.4)	4.9 (4.4; 5.5)

^1^Apolipoprotein MOrtality RISk (AMORIS).

^2^Metabolic syndrome and Cancer project (Me-Can).

^3^Anaplastic astrocytoma and glioblastoma.

^4^IQR: interquartile range.

^5^Age at glioma diagnosis (event), death, emigration, or end of follow-up (censored); in the Me-Can cohort observations are also censored at the time of diagnosis of any cancer.

Hazard ratios and trend test *P*-values in Table 2 are also similar for both sexes combined in the AMORIS and Me-Can cohorts, although these *P*-values primarily reflect the hazard ratios of the highest level of glucose (≥6.1 mmol/L) (AMORIS HR = 0.59, 95% CI 0.42 to 0.84; Me-Can HR = 0.58, 95% CI 0.32 to 1.04). Results restricted to high grade glioma (anaplastic astrocytoma and glioblastoma; Supplemental Table 1) do not differ substantially from those in Table 2.

**Table 2:** Associations between pre-diagnostic blood glucose levels and glioma by cohort and gender.

Glucose Level^2^	Hazard ratios^1^ (95% confidence intervals)					*P*_trend_^3^
	<4.6 mmol/L	4.6– < 5.1 mmol/L	5.1– < 5.6 mmol/L	5.6– < 6.1 mmol/L	≥6.1 mmol/L	
**AMORIS** ^**4**^ **cohort**						
Men	1.00 (ref)	0.92 (0.71 to 1.19)	0.83 (0.61 to 1.11)	0.79 (0.53 to 1.16)	0.66 (0.45 to 0.98)	0.03
Cases, n	103	128	85	36	38	
Women	1.00 (ref)	0.99 (0.77 to 1.36)	1.48 (1.02 to 2.14)	0.88 (0.48 to 1.63)	0.39 (0.17 to 0.86)	0.02
Cases, n	74	66	54	13	7	
Total	1.00 (ref)	0.96 (0.78 to 1.18)	1.02 (0.81 to 1.29)	0.81 (0.58 to 1.12)	0.59 (0.42 to 0.84)	0.002
Cases, n	177	194	139	49	45	
Person-years at risk	2,566,979	2,539,673	1,423,518	526,040	449,604	
**Me-Can** ^**5**^ **cohort**						
Men	1.00 (ref)	0.94 (0.57 to 1.57)	0.77 (0.43 to 1.39)	0.75 (0.35 to 1.58)	0.51 (0.22 to 1.19)	0.10
Cases, n	34	29	19	10	7	
Women	1.00 (ref)	1.03 (0.59 to 1.79)	1.06 (0.58 to 1.94)	0.74 (0.33 to 1.65)	0.67 (0.29 to 1.53)	0.24
Cases, n	28	24	21	9	8	
Total	1.00 (ref)	0.97 (0.66 to 1.41)	0.89 (0.58 to 1.35)	0.73 (0.42 to 1.27)	0.58 (0.32 to 1.04)	0.04
Cases, n	62	53	40	19	15	
Person-years at risk	956,856	687,385	536,687	268,187	218,250	

^1^Hazard ratios adjusted for age at lab test, date of lab test, fasting, triglycerides, cholesterol, and gender (totals only); with separate baseline hazards for time before glioma diagnosis and age at glioma diagnosis and sub-cohort in the Me-Can data set.

^2^Fourth and fifth lower cut points are based on American Diabetes Association (54) and World Health Organization (55) definition of pre-diabetes and impaired fasting glucose respectively

^3^Linear contrasts (ANOVA, F-test).

^4^Apolipoprotein and MOrtality RISk (AMORIS).

^5^Metabolic syndrome and Cancer project (Me-Can); number of cases differs from those in Table 1 due to missing values of triglycerides, cholesterol, and glucose.

Hazard ratios and trend test P-values produced by the sub-distribution hazards models (Supplemental Table 2) in the AMORIS cohort, are similar to those in the standard Cox models (Table 2) thus providing no evidence of competing risks. However, in the Me-Can cohort while hazard ratios (Supplemental Table 2) are similar to those in Table 2, *P*-values differ slightly. Taken together, however, these results indicate that competing risks are probably not driving our findings.

Pre-diagnostic diabetes is strongly inversely associated with glioma in the AMORIS cohort, (HR = 0.30, 95% CI 0.17 to 0.63), although it is based on only 12 glioma cases (2.0% of total glioma cases), 11 of which were found among men. There were 39,404 people (7.5% of the total non-cases) diagnosed with diabetes among the non-cases, men accounted for 25,392 of these diagnoses. Information on pre-diagnostic diabetes diagnoses was not available for the Me-Can cohort.

Table 3 shows hazard ratios by the time between the first blood glucose test and glioma diagnosis. These hazard ratios represent the change in the glioma hazard rate with a one unit change in blood glucose level. For example, among men whose blood was collected within the year before the end of follow-up, the hazard ratio based on the AMORIS data (HR = 0.78, 95% CI 0.65 to 0.94) indicates that glioma rates decrease 22% per unit increase of the standardised natural log of blood glucose. In addition, the trend test for both sexes combined indicates the presence of a linear trend (*P*_trend_ = 0.04) over the levels of time before diagnosis. Thus the time of blood draw modifies the effect of glucose on the glioma rate, that is, there is a statistically significant interaction between time and glucose. There is no evidence of a similar effect of time before diagnosis in the Me-Can cohort. In addition, in an exploratory analysis of within-person time trends in 51 Me-Can patients with multiple blood glucose measurements, we found no trends by year before glioma diagnosis.

**Table 3:** Associations between pre-diagnostic blood glucose level^1^ and glioma by time between first blood glucose test and glioma diagnosis by cohort and gender.

Time from first test to glioma diagnosis	Hazard ratios^2^ (95% confidence intervals)				*P*_trend_^3^
	<1 year	1– < 5 years	5– < 10 years	10– ≤ 15 years	
**AMORIS** ^**4**^ **cohort**					
Men	0.78^5^ (0.65 to 0.94)	0.86 (0.74 to 0.99)	1.03 (0.87 to 1.21)	0.89 (0.75 to 1.05)	0.07
Cases, n	26	112	130	122	
Women	0.84 (0.58 to 1.20)	1.00 (0.81 to 1.22)	0.80 (0.62 to 1.05)	1.04 (0.79 to 1.36)	0.48
Cases, n	22	61	76	55	
Total	0.78 (0.66 to 0.93)	0.88 (0.78 to 1.00)	0.97 (0.83 to 1.13)	0.94 (0.81 to 1.09)	0.04
Cases, n	48	173	206	177	
Person-years at risk	1,516	42,459	158,410	7,303,429	
**Me-Can cohort** ^**6**^					
Men	1.06 (0.59 to 1.90)	0.73 (0.50 to 1.06)	0.79 (0.55 to 1.12)	1.21 (0.87 to 1.69)	0.77
Cases, n	7	21	41	30	
Women	0.92 (0.45 to 1.87)	0.86 (0.60 to 1.23)	0.81 (0.57 to 1.16)	0.85 (0.51 to 1.41)	0.89
Cases, n	9	25	39	17	
Total	0.99 (0.63 to 1.56)	0.79 (0.60 to 1.02)	0.79 (0.61 to 1.02)	1.07 (0.80 to 1.44)	0.82
Cases, n	16	46	80	47	
Person-years at risk	754	122,003	479,639	2,064,969	

^1^Glucose was transformed to its standardised log (i.e. mean = 0, standard deviation = 1); one unit of this transformed value equals one standard deviation.

^2^Hazard ratios adjusted for age at lab test, date of lab test, fasting, triglycerides, cholesterol, and gender (totals only); with separate baseline hazards for age at glioma diagnosis and for sub-cohort in the Me-Can data set.

^3^Linear contrasts (ANOVA, F-test).

^4^Apolipoprotein and MOrtality RISk (AMORIS) cohort.

^5^A hazard ratio of 0.78 among men whose blood was drawn ≤1 year before diagnosis indicates that the glioma rate decreases 22% per unit increase of the standardised natural log of blood glucose.

^6^Metabolic syndrome and Cancer project (Me-Can); number of cases differs from Table 1 due to missing values of triglycerides, cholesterol, and glucose.

Age at glioma diagnosis (Table 4) significantly (*P* < 0.05) modifies the association between glucose and glioma risk among men older than age 70 years in the Me-Can cohort (Table 4, Me-Can: HR = 0.50, 95% CI 0.29 to 0.85), but does not in the AMORIS cohort (AMORIS: HR = 0.84, 95% CI 0.64 to 1.11). Age trends are of borderline statistical significance.

**Table 4:** Associations between pre-diagnostic blood glucose level^1^ and glioma by age at glioma diagnosis, cohort and gender.

Age at diagnosis	Hazard ratios^2^ (95% confidence intervals)				*P*_trend_^3^
	≤50 years	>50–≤60 years	>60–≤70 years	>70 years	
**AMORIS** ^**4**^ **cohort**					
Men	1.01 (0.87 to 1.79)	0.79 (0.68 to 0.92)	0.80 (0.69 to 0.93)	0.84 (0.64 to 1.11)	0.08
Cases, n	126	104	126	34	
Women	0.94 (0.69 to 1.28)	0.94 (0.73 to 1.19)	0.78 (0.60 to 0.98)	1.07 (0.82 to 1.42)	0.93
Cases, n	56	68	64	26	
Total	0.98 (0.85 to 1.13)	0.81 (0.71 to 0.92)	0.81 (0.72 to 0.92)	0.93 (0.76 to 1.15)	0.10
Cases, n	182	172	190	60	
Person-years at risk	2,211,354	1,995,967	1,816,781	1,481,712	
**Me-Can** ^**5**^ **cohort**					
Men	0.97 (0.61 to 1.54)	1.08 (0.82 to 1.42)	0.72 (0.47 to 1.10)	0.50^6^ (0.29 to 0.85)	0.06
Cases, n	20	50	22	7	
Women	0.78 (0.43 to 1.42)	0.77 (0.51 to 1.17)	0.87 (0.61 to 1.24)	0.72 (0.41 to 1.25)	0.94
Cases, n	18	29	33	10	
Total	0.87 (0.60 to 1.27)	0.96 (0.76 to 1.22)	0.81 (0.62 to 1.07)	0.60 (0.40 to 0.90)	0.26
Cases, n	38	79	55	17	
Person-years at risk	900,761	662,854	663,638	440,112	

^1^Glucose was transformed to its standardised log (i.e. mean = 0, standard deviation = 1); one unit of this transformed value equals one standard deviation.

^2^Hazard ratios adjusted for age at lab test, date of lab test, fasting, triglycerides, cholesterol, and gender (totals only); with separate baseline hazards for age at glioma diagnosis and for sub-cohort in the Me-Can data set.

^3^Linear contrasts (ANOVA, F-test).

^4^Apolipoprotein and MOrtality RISk (AMORIS).

^5^Metabolic syndrome and Cancer project (Me-Can); number of cases differs from Table 1 due to missing values of triglycerides, cholesterol, and glucose.

^6^A hazard ratio of 0.50 among men whose blood was drawn when they were older than 70 years indicates that the glioma rate decreases 50% per unit increase of the standardised natural log of blood glucose.

## Discussion

We found inverse associations between pre-diagnostic blood glucose levels and glioma risk in both the AMORIS and Me-Can cohorts, and between diabetes and glioma in the AMORIS cohort. This blood glucose - glioma association was modified by time between the first blood glucose test and glioma diagnosis in the AMORIS cohort and possibly by age at glioma diagnosis in the Me-Can cohort. In addition, we found no evidence of competing risks.

Our blood glucose dose-response findings are similar to those reported for high grade and total glioma by Edlinger *et al*.^10^, using four additional sub-cohorts of the Me-Can study, and by Seliger *et al*.^7^. The inverse association between diabetes and glioma, in the AMORIS cohort, is also consistent with the majority of the previous literature^7, 8^.

The initial examination of the time and age interactions was hypothesis driven, as noted in the introduction, rather than based on statistical significance alone (see Statistical methods). In addition, the initial significance tests reflected the statistical significance of a single interaction term (stratified on the second time-related term). These tests are not as meaningful as the interaction analyses presented in Tables 3 and 4 because they mask time and age-specific trends. The significant and borderline significant trends in these tables show time interactions in the AMORIS cohort and age interactions in both cohorts. As described in the introduction, both time and age interactions may represent biologically meaningful effects. To assess their validity, these results require confirmation in subsequent studies.

The sub-distribution hazards model indicates the absence of competing risks; however, the weakness of this analytic approach is that although people who die are retained in risk sets after their deaths, the number of people diagnosed with glioma remains the same^22, 23^. Given this limitation, the strong inverse age-blood glucose association among people older than age 70 years in the Me-Can cohort may indicate the presence of competing risks from hyperglycaemia-associated deaths. However if our findings are attributable to competing risks one would expect the inverse diabetes-cancer relationship to be observed at cancer sites other than glioma. In fact, we see the opposite pattern. Tumours at most sites are positively associated with blood glucose level^3, 24–26^ with the known exceptions of meningioma in one study^27^ but not in another^9^ and prostate cancer^18, 28^.

Limitations of our study include the lack of sufficient information on body mass index, blood pressure and high and low density lipoprotein in the AMORIS cohort to include these variables in our regression models. These variables are associated with both diabetes^29^ and glioma^10^ and therefore qualify as potentially confounding variables. Also, there are several differences between the AMORIS and Me-Can cohorts that may affect comparisons. Serum glucose was measured in the AMORIS cohort while plasma glucose was measured in the Me-Can cohort. However Frank *et al*.^30^ found no physiologically relevant differences between these two methods of measuring circulating glucose. Another difference between these cohorts is that non-fasting serum glucose values were included in the AMORIS cohort but were not included in the Me-Can cohort although fasting and non-fasting glucose values in the AMORIS cohort were similar. In addition, Me-Can study participants were censored after diagnosis of any malignant tumour while AMORIS cohort participants were not. Despite these differences, cohort dose-response findings for the two cohorts are remarkably similar. Whether conflicting estimates of time and age interactions with blood glucose levels produced by the two cohorts are attributable to their differences in design is not known.

There are several potential mechanisms accounting for the inverse association between hyperglycaemia and glioma. The commonly prescribed anti-diabetic medication, metformin, inhibits glioblastoma cell proliferation and migration^31^ and may therefore reduce glioma risk. However, a large case-control study found no clear evidence of this association^7^.

Adverse effects of hyperglycaemia and diabetes that may paradoxically reduce glioma risk include diabetes-related reduction of bioavailable testosterone levels^7, 32^. Another possibility is the fact that both impaired glucose tolerance^33^ and diabetes^34–36^ lead to the shortening of telomeres (that is, the region at each end of a chromosome which prevents deterioration but shortens with each cell replication). The highest glioma risk alleles near the TERC and TERT gene are associated with long telomeres^37^ thus providing a potential rationale for our findings. The problem with this line of reasoning, however, is that one would expect hyperglycaemia to be inversely related to malignant melanoma, the risk of which is also positively associated with telomere length^38^, but it is not^39^.

Hyperglycaemia induces apoptosis and inhibits progression of neural stem cells^40^. To the extent that glioblastoma stem cells are related to neural stem cells^41^ hyperglycaemia may confer protection against glioblastoma. Another potential mechanism is inhibition of cerebral circulation associated with diabetes^42, 43^ causing reduced glioma risk^44^. In addition, lower levels of insulin-like growth factor 1 (IGF-1) are found in diabetic than in non-diabetic serum^45^ and tumour-associated macrophage derived IGF-1 may drive glioma recurrence^46^. These proposed mechanisms may work together or separately.

Overall, our findings provide further evidence of an inverse association between hyperglycaemia, diabetes and glioma. In contrast, evidence for the presence of the Warburg effect^11^ was mixed with the AMORIS data suggesting an inverse association during the year before glioma diagnosis and the Me-Can data showing no such association. Further investigation of a pre-diagnostic Warburg effect would be worthwhile, as would examination of associations between blood glucose level and glioma by genetic subtypes of type 2 diabetes^47^ and glioma^48^.

## Methods

The AMORIS and Me-Can cohorts have been described in detail elsewhere^49–51^. Here we briefly summarise this information.

### AMORIS cohort

This Swedish cohort links records from the Central Automation Laboratory (CALAB, Stockholm, Sweden) database (1985–1996) to those of the National Cancer Registry, the National Patient Register, the Cause of Death Register, and the National Register of Emigration. Endpoint ascertainment is based on the Swedish National Cancer Registry and therefore includes all glioma diagnoses among Swedish residents. Outcome data used in the present analyses are current as of December 31^st^, 2011.

The AMORIS cohort consists of 812,073 participants of all ages with 529,123 having been tested for serum glucose during their first lab visit. People under age 18 years old at the time of glioma diagnosis (event of interest) or death, emigration, or the end of follow-up (outcomes leading to censoring) were excluded from the study as their tumours may differ from those found in adults^52^. We then excluded 528 people diagnosed with meningioma (defined by pathology-anatomy diagnosis (PAD) codes (WHO/HS/CANC/24.1, Histology Code) 461 and 466) and 15 with ependymoma (PAD codes 481, 485). These additional exclusions reduced the sample to 528,580 participants, 604 of whom were subsequently diagnosed with glioma (PAD codes 475 (n = 128) and 476 (n = 476) and the International Classification of Disease 7^th^ Edition (ICD-7) code 193) (see Supplemental Table 3). This project has been approved by the Regional Ethical committee at the Karolinska Institutet, Stockholm, Sweden (Registration number 2010/1:7). All the study data were de-identified and the ethical committee approved the project without collecting any additional informed consent. The project complied with the requirements of the Declaration of Helsinki.

Information on diabetes in the AMORIS cohort was based on the National Diabetes Register^53^ established in 1996 and the National Patient Register which began including outpatient diagnoses in 2001. The AMORIS study began in  1985 and the data set used in the present study ended in 2011 so information on diabetes was not complete, but presumably equally incomplete for glioma cases and non-cases. To partially address the absence of information on diabetes before 1996, we also classified individuals in our sample as diabetics if they had fasting serum glucose levels ≥ 7.0 mmol/L.

### Me-Can cohort

Seven population-based sub-cohorts were originally included in the Me-Can study, however in the present investigation we used data from only the Austrian and the two Swedish sub-cohorts^51^. In brief, these sub-cohorts contained data on 269,365 participants, ranging from 19 to 96 years of age at baseline (between 1974 and 2005) without a known tumour, after exclusion of 2,711 non-fasting participants. Data included measured height, weight and blood pressure, as well as levels of fasting plasma glucose, triglycerides and total cholesterol. For this study, each individual’s baseline data were taken from the first (or only) health examination with complete or near-complete data. Nationwide cancer and cause-of-death registries were used to follow participants. Glioma diagnoses were based on PAD codes 475 (n = 21) and 476 (n = 96) and ICD-7 193 codes in the Swedish sub-cohorts. In the Austrian sub-cohort these diagnoses were identified using ICD-7 193 and histology codes 93803, 93813, 93823, 94003, 94013, 94113, 94203, 94213, 94403, 94503, and 94513 from ICD-O1 (see Supplemental Table 3). In this sub-cohort meningioma cases were not identified and are thus included among the non-cases. Ascertainment of endpoints in the Swedish sub-cohorts is based on the Swedish Cancer Registry and is  complete. The Austrian sub-cohort is regularly linked to the regional cancer registry with high coverage and completeness. The study was approved by The Research Review Board of Umeå, Sweden, the Regional Committee for Medical and Health Research Ethics and the Ethikkommission of the Land Vorarlberg, Austria. Participants in Sweden and in Austria provided written informed consent to participate in the studies. All study data were de-identified.

The association between blood glucose and glioma risk has been previously reported using all seven population-based Me-Can sub-cohorts^10^ (we use only three of these in the present study). Here the exposure categories are different to allow comparison with the AMORIS cohort. More importantly, we evaluated time and age interactions and competing risks, which were not examined previously. Also, an exploratory analysis was performed using a subgroup with repeated exposure measurements.

### Statistical methods

In our multivariable time-dependent Cox regression models, study participants were included in the analysis at their age at the time of their first blood glucose test (left-truncation) and exited the analysis at the age they were diagnosed with glioma or died, emigrated or when follow-up ended (whichever event came first). In addition, in the Me-Can cohort participants were also censored at the time of diagnosis of any malignant tumour. Follow-up time was restricted to a maximum of 15 years from the initial blood glucose test because we were interested in the hypothetical effect of the preclinical tumour on glucose levels (reverse causality) as well as the possible effects of blood glucose levels on glioma risk.

In the initial glucose dose-response analysis the first three lower cut points for the blood glucose variable were based on those in a previous paper using AMORIS cohort data^18^, the lowest cut point for the fourth glucose level (5.6 mmol/L) is, according to the American Diabetes Association^54^, the lowest level of blood glucose that indicates pre-diabetes, while the lower cut point for the fifth glucose level (6.1 mmol/L) is the lowest blood glucose value designated by the World Health Organization as an indication of impaired fasting glucose^55^. We used these cut points rather than quintiles because of their clinical significance.

Our time- dependent Cox regression models were adjusted for age at the time of the first blood glucose test, date at the time of the blood glucose test, fasting status, triglycerides, cholesterol levels, and gender. Using data from the AMORIS cohort, we found that when characterised by a single interaction term and stratified on the other time-related variable, the association between blood glucose and glioma varied with the time of the blood draw before glioma diagnosis (HR = 0.98, 95% CI = 0.96 to 1.00, *P* = 0.03) and age at glioma diagnosis (HR = 1.00, 95% CI = 1.00 to 1.01, *P* = 0.11). We therefore used the strata statement in PROC PHREG (SAS, version 9.4) and stratified these regression models by both time before diagnosis and age at diagnosis thus producing separate baseline hazards for each time and age-specific stratum^56^. For consistency, the Me-Can data were also stratified by sub-cohort, but not adjusted for fasting status since the few non-fasting participants were not included in the data set. We tested dose-response trends over time and age using linear contrasts, analysis of variance, and F-tests^57^.

In addition to estimating the overall association between blood glucose level and glioma, accounting for the modifying effects of time and age, we also estimated these time-related effects. As noted in the introduction above, these analyses were driven by theory, in addition to the initial statistical significance of the time-glioma interaction and borderline significance of the age interaction in the AMORIS data. Instead of using five categories of blood glucose, as we did in the dose-response Cox regression models, we analysed blood glucose level as a continuous variable within each of four categories of years before glioma diagnosis (≤1, 1–<5, 5–<10, 10–<15) and four categories of age in years at glioma diagnosis (≤50, >50–≤60, >60–≤70, >70). To avoid multicollinearity, we used two separate time-dependent Cox regression models; one representing time interactions and a second representing age interactions. To address the potential problem of the influence of outliers on the results, we used natural logarithms of the blood glucose values and standardised them to a mean of zero and a standard deviation of one. In addition, we accounted for that time-related variable which was not included in the model using the strata statement in PROC PHREG (SAS, version 9.4).

All Me-Can cohort study participants fasted before their blood was drawn; however only 56% of the AMORIS study participants did so. We therefore analysed AMORIS glucose dose-response relationships separately by fasting status. We found that fasting and non-fasting glucose dose-response relationships were similar (Supplemental Table 4) allowing us to combine data from these two groups.

Using the Me-Can data set, we conducted an exploratory analysis of the 51 glioma cases with multiple measurements to determine whether glucose values ≤7.0 mmol/L varied with the number of measurements or time between the blood glucose test and glioma diagnosis.

To find out whether competing risks affected our findings, in addition to estimating the potentially modifying effects of age at glioma diagnosis on the blood glucose-glioma association, we used the sub-distribution hazards regression model (SAS, version 9.4). Hazard ratios based on the latter model are those which would have been found had individuals who were at risk of glioma when they died remained in the population but were not subsequently diagnosed with glioma^22, 23^.

All analyses were conducted using SAS statistical software, version 9.4 (SAS Institute Inc, Cary, NC).

## Electronic supplementary material

Supplemental Tables
