# Supplementary material for: Associations between prediagnostic blood glucose levels, diabetes, and glioma
Source: Sci Rep. 2017 May 3;7:1436. doi: 10.1038/s41598-017-01553-2 (PMC5431098; doi:10.1038/s41598-017-01553-2)
Supplement: Supplementary file 1 — Supplemental Tables [file 41598_2017_1553_MOESM1_ESM.pdf]

Associations between prediagnostic blood glucose levels, diabetes, and glioma

Judith Schwartzbaum*^, Michael Edlinger*^, Victoria Zigmont, Pär Stattin, Grzegorz A. Rempala, Gabriele Nagel, Niklas Hammar, Hanno Ulmer, Bernhard Föger, Göran Walldius, Jonas Manjer, Håkan Malmström, Maria Feychting

^ contributed equally

* corresponding author [ja.schwartzbaum@gmail.com]

* corresponding author [michael.edlinger@i-med.ac.at]

Supplemental Table 1. Association between pre-diagnostic blood glucose and high grade glioma[1] by cohort and gender

	Hazard ratios[2] (95% confidence intervals)					
**Glucose Level[3]**	**<4.6 mmol/L**	**4.6-<5.1 mmol/L**	**5.1-<5.6 mmol/L**	**5.6-<6.1 mmol/L**	**≥6.1mmol/L**	**P_{trend}[4]**
			**AMORIS[5] cohort**			
**Men**	1.00 (ref)	0.95 (0.70 to 1.29)	0.84 (0.60 to 1.17)	0.88 (0.58 to 1.36)	0.69 (0.45 to 1.07)	0.10
**Cases, n**	75	98	65	31	32	
**Women**	1.00 (ref)	1.10 (0.76 to 1.60)	1.61 (1.07 to 2.43)	0.86 (0.43 to 1.72)	0.48 (0.21 to 1.07)	0.08
**Cases, n**	56	56	46	10	7	
**Total**	1.00 (ref)	1.01 (0.80 to 1.28)	1.07 (0.83 to 1.40)	0.88 (0.61 to 1.26)	0.64 (0.44 to 0.94)	0.02
**Cases, n**	131	154	111	41	39	
**Person-years at risk**	2,566,668	2,539,414	1,423,364	525,999	449,557	
			**Me-Can[6] cohort**			
**Men**	1.00 (ref)	0.90 (0.52 to 1.55)	0.63 (0.33 to 1.23)	0.84 (0.39 to 1.81)	0.51 (0.20 to 1.26)	0.17
**Cases, n**	30	24	14	10	6	
**Women**	1.00 (ref)	0.87 (0.45 to 1.68)	1.06 (0.53 to 2.10)	0.50 (0.18 to 1.40)	0.72 (0.29 to 1.80)	0.28
**Cases, n**	21	16	17	5	7	
**Total**	1.00 (ref)	0.87 (0.57 to 1.32)	0.80 (0.50 to 1.28)	0.68 (0.37 to 1.26)	0.60 (0.31 to 1.14)	0.09
**Cases, n**	51	40	31	15	13	
**Person-years at risk**	956,856	687,385	536,687	268,187	218,250	

1. Anaplastic astrocytoma and glioblastoma
2. Hazard ratios adjusted for age at lab test, date of lab test, fasting, triglycerides, cholesterol, and gender (totals only); with separate baseline hazards for time before glioma diagnosis and age at glioma diagnosis and sub-cohort only in the Me-Can data set
3. Fourth and fifth lower cut points are based on American Diabetes Association (54) and World Health Organization (55) definition of pre-diabetes and impaired fasting glucose respectively
4. Linear contrasts (ANOVA, F-test)
5. Apolipoprotein and MOrtality RISk (AMORIS)
6. Metabolic syndrome and Cancer project (Me-Can); number of cases differs Table 1 due to missing values of triglycerides, cholesterol, and glucose

Supplemental Table 2. Sub-distribution[1] hazards regression analysis of the association between pre-diagnostic blood glucose levels and glioma by cohort and gender

	Hazard ratios[2] (95% confidence intervals)					
**Glucose Level[3]**	**<4.6 mmol/L**	**4.6-<5.1 mmol/L**	**5.1-<5.6 mmol/L**	**5.6-<6.1 mmol/L**	**≥6.1 mmol/L**	**P_{trend}[4]**
			**AMORIS[5] cohort**			
**Men**	1.00 (ref)	0.96 (0.79 to 1.25)	0.87 (0.64 to 1.18)	0.78 (0.53 to 1.16)	0.64 (0.43 to 0.97)	0.02
**Cases, n**	103	128	85	36	38	
**Women**	1.00 (ref)	0.99 (0.71 to 1.39)	1.49 (1.04 to 2.15)	0.87 (0.48 to 1.59)	0.37 (0.16 to 0.82)	0.02
**Cases, n**	74	66	54	13	7	
**Total**	1.00 (ref)	0.98 (0.79 to 1.20)	1.05 (0.83 to 1.33)	0.82 (0.59 to 1.13)	0.58 (0.41 to 0.83)	0.002
**Cases, n**	177	194	139	49	45	
**Person-years at risk**	2,566,979	2,539,673	1,423,518	526,040	449,604	
			**Me-Can[6] cohort**			
**Men**	1.00 (ref)	0.93 (0.57 to 1.52)	0.72 (0.41 to 1.27)	0.69 (0.34 to 1.41)	0.50 (0.21 to 1.16)	0.08
**Cases, n**	34	29	19	10	7	
**Women**	1.00 (ref)	1.10 (0.64 to 1.90)	1.18 (0.66 to 2.12)	0.93 (0.44 to 1.98)	0.84 (0.37 to 1.95)	0.59
**Cases, n**	28	24	21	9	8	
**Total**	1.00 (ref)	1.01 (0.70 to 1.45)	0.91 (0.61 to 1.37)	0.80 (0.47 to 1.34)	0.64 (0.36 to 1.17)	0.10
**Cases, n**	62	53	40	19	15	
**Person-years at risk**	956,856	687,385	536,687	268,187	218,250	

1. Based on cumulative incidence to identify the presence of competing risks (23)
2. Hazard ratios adjusted for age at lab test, date of lab test, fasting, triglycerides, cholesterol, and gender (totals only); with separate baseline hazards for time before glioma diagnosis and age at glioma diagnosis and sub-cohort only in the Me-Can data set
3. Fourth and fifth lower cut points are based on American Diabetes Association (54) and World Health Organization (55) definition of pre-diabetes and impaired fasting glucose respectively
4. Linear contrasts (ANOVA, F-test)
5. Apolipoprotein and MOrtality RISk (AMORIS)
6. Metabolic syndrome and Cancer project (Me-Can); number of cases differs Table 1 due to missing values of triglycerides, cholesterol, and glucose

Supplemental Table 3. Histologic codes used to identify glioma cases in the AMORIS[1] and Me-Can[2] cohorts

Histology	Code	AMORIS cohort n of cases	Me-Can cohort n of cases
		Swedish cases	
Malignant neoplasms of brain and other parts of the nervous system	ICD-7 193	604	117
Low grade	PAD 475	128	21
High grade	PAD 476	476	96
			Austrian cases
Malignant neoplasms of brain and other parts of the nervous system	ICD-7 193	.	91
Low grade	ICD-O1 93823	.	4
Low grade	ICD-O1 94003	.	9
Low grade	ICD-O1 94113	.	2
Low grade	ICD-O1 94203	.	1
Low grade	ICD-O1 94213	.	4
Low grade	ICD-O1 94503	.	3
High grade	ICD-O1 93803	.	1
High grade	ICD-O1 93813	.	1
High grade	ICD-O1 94013	.	6
High grade	ICD-O1 94403	.	59
High grade	ICD-O1 94513	.	1

1. Apolipoprotein and MOrtality RISk (AMORIS)
2. Metabolic syndrome and Cancer project (Me-Can)

Supplemental Table 4. Associations in the AMORIS[1] cohort between fasting and non-fasting pre-diagnostic blood glucose levels and glioma by gender

	Hazard ratios[2] (95% confidence intervals)					
**Glucose Level[3]**	**<4.6 mmol/L**	**4.6-<5.1 mmol/L**	**5.1-<5.6 mmol/L**	**5.6-<6.1 mmol/L**	**≥6.1 mmol/L**	P_{trend}[4]
			**Fasting**			
**Men**	1.00 (ref)	0.89 (0.63 to 1.24)	0.79 (0.54 to 1.15)	0.49 (0.28 to 0.88)	0.81 (0.50 to 1.32)	0.08
**Cases, n**	60	81	56	15	27	
**Women**	1.00 (ref)	1.03 (0.67 to 1.57)	1.09 (0.65 to 1.18)	1.02 (0.48 to 2.17)	0.39 (0.13 to 1.13)	0.11
**Cases, n**	45	45	24	9	4	
**Total**	1.00 (ref)	0.95 (0.73 to 1.23)	0.87 (0.65 to 1.22)	0.62 (0.39 to 0.71)	0.72 (0.47 to 1.11)	0.03
**Cases, n**	105	126	80	24	31	
**Person-years at risk**	1,408,888	1,493,242	821,735	284,303	211,487	
			**Non-Fasting**			
**Men**	1.00 (ref)	0.94 (0.61 to 1.44)	0.90 (0.55 to 1.46)	1.39 (0.81 to 2.41)	0.47 (0.23 to 0.95)	0.15
**Cases, n**	43	47	29	21	11	
**Women**	1.00 (ref)	0.88 (0.49 to 1.56)	2.10 (1.21 to 3.63)	0.64 (0.22 to 1.89)	0.34 (0.09 to 1.19)	0.08
**Cases, n**	29	21	30	4	3	
**Total**	1.00 (ref)	0.95 (0.68 to 1.33)	1.28 (0.90 to 1.84)	1.15 (0.72 to 1.85)	0.44 (0.24 to 0.80)	0.03
**Cases, n**	72	68	59	25	14	
**Person-years at risk**	1,158,090	1,046,431	601,783	241,737	238,117	

1. Apolipoprotein and MOrtality RISk (AMORIS)
2. Hazard ratios adjusted for age at lab test, date of lab test, triglycerides, cholesterol, and gender (totals only); with separate baseline hazards for time before glioma diagnosis and age at glioma diagnosis
3. Fourth and fifth lower cut points are based on American Diabetes Association (54) and World Health Organization (55) definition of pre-diabetes and impaired fasting glucose respectively
4. Linear contrasts (ANOVA, F-test)
